# Pharmacological activities and mechanisms of action of Pogostemon cablin Benth: a review

**DOI:** 10.1186/s13020-020-00413-y

**Published:** 2021-01-07

**Authors:** Chen Junren, Xie Xiaofang, Li Mengting, Xiong Qiuyun, Li Gangmin, Zhang Huiqiong, Chen Guanru, Xu Xin, Yin Yanpeng, Peng Fu, Peng Cheng

**Affiliations:** 1 Chengdu University of Traditional Chinese Medicine, Chengdu, 610075 China; 2 State Key Laboratory of Traditional Chinese Medicine Resources in Southwest China, Chengdu, 610075 China; 3 West China School of Pharmacy, Sichuan University, 17 South Renmin Rd, 610065 Chengdu, China; 4 School of Pharmacy, Chengdu University of Traditional Chinese Medicine, 37 Shierqiao Road, Jinniu District, Chengdu, 611137 China

**Keywords:** *Pogostemon cablin* Benth. (patchouli), Mechanisms, Biological activities, Patchouli alcohol

## Abstract

Patchouli (“Guanghuoxiang”) or scientifically known as *Pogostemon cablin* Benth, belonging to the family Lamiaceae, has been used in traditional Chinse medicine (TCM) since the time of the Eastern Han dynasty. In TCM theory, patchouli can treat colds, nausea, fever, headache, and diarrhea. Various bioactive compounds have been identified in patchouli, including terpenoids, phytosterols, flavonoids, organic acids, lignins, glycosides, alcohols, pyrone, and aldehydes. Among the numerous compounds, patchouli alcohol, β-patchoulene, patchoulene epoxide, pogostone, and pachypodol are of great importance. The pharmacological impacts of these compounds include anti-peptic ulcer effect, antimicrobial effect, anti-oxidative effect, anti-inflammatory effect, effect on ischemia/reperfusion injury, analgesic effect, antitumor effect, antidiabetic effect, anti-hypertensive effect, immunoregulatory effect, and others.For this review, we examined publications from the previous five years collected from PubMed, Web of Science, Springer, and the Chinese National Knowledge Infrastructure databases. This review summarizes the recent progress in phytochemistry, pharmacology, and mechanisms of action and provides a reference for future studies focused on clinical applications of this important plant extract.

## Introduction

**Fig. 1:**
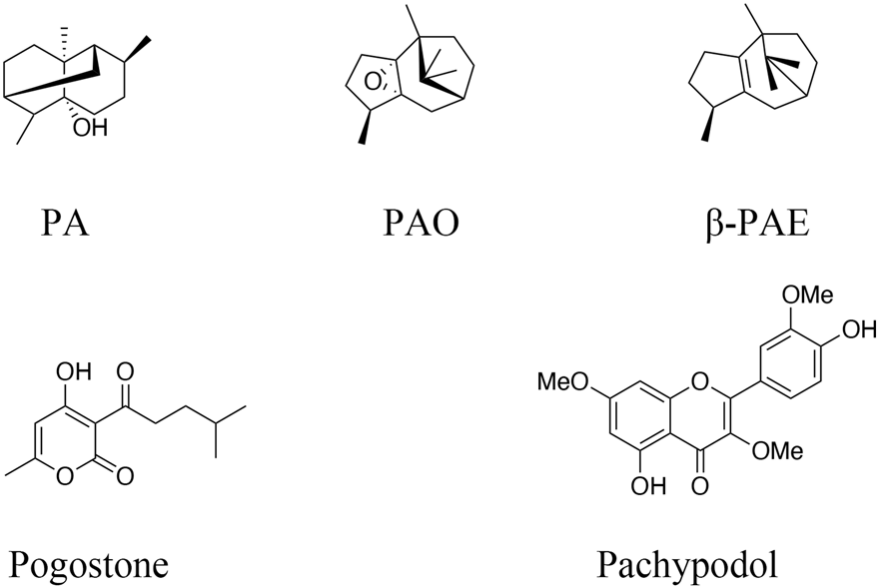
The chemical structures of PA, β-PAE, PAO, pogostone, and pachypodol

*Pogostemon cablin* Benth., also known as patchouli, or “Guanghuoxiang” in traditional Chinese medicine (TCM), is a member of the Lamiaceae family of flowering plants and has been used to treat colds, nausea, fever, headache, and diarrhea [1]. Guanghuoxiang is among the raw materials used in formulations of numerous famous Chinese patent medicines, including Huoxiang Zhengqi Koufuye (oral liquid) and Baoji Pian (tablets). Huoxiang Zhengqi Koufuye can be used to treat gastrointestinal diseases, while Baoji Pian is mainly used for common cold with summer-heat and dampness syndrome in TCM [2–4]. Previous research revealed that patchouli was composed of a variety of chemical substances, including monoterpenoids, triterpenoids, sesquiterpenoids, phytosterols, flavonoids, organic acids, lignins, glycosides, alcohols, pyrone, and aldehydes [5]. Given its multicomponent nature, patchouli has been found to promote numerous pharmacological activities, and has been shown to protect against inflammation [6], microorganisms [7, 8], tumors [9], aging [10], and oxidation [11]. Moreover, patchouli and its extracts exert remarkable beneficial effects that promote the healthy functioning of organs and tissues. Among these findings, patchouli extracts have been shown to protect against gastrointestinal infection with *Helicobacter pylori* [12] and ulcers [13]; they can also suppress adipogenesis and fat accumulation in adipocytes [14], alleviate ischemia/reperfusion-induced brain injury [15], and prevent atherosclerosis [16]. Based on previous review, it can be concluded that patchouli alcohol (PA), β-patchoulene (β-PAE), patchoulene epoxide (PAO), pogostone, and pachypodol are the material basis for patchouli to exert therapeutic effects. As a significant ingredient in patchouli, PA has been most intensively studied in the pharmacological effects including anti-inflammatory effect, anti-apoptotic effect, anti-oxidative effect, anti-tumor effect, and others. Recently, some new pharmacological effects of PA have been explored, a research in 2019 has illustrated that PA could suppress adipogenesis and fat accumulation in adipocytes to prevent obesity [17]. Another study in the same year demonstrated that PA could exert analgesic effect by regulating opioid receptors [18]. Furthermore, PA could exert an intensively vasorelaxant effect as a Ca^2+^ antagonist [19]. In addition to PA, pogostone has been reported to possess gastroprotective, anti-photoaging, and antimicrobial properties. Furthermore, a research in 2017 found that pogostone exerted the antitumor activity [20], and another study in 2019 revealed that pogostone could protect lung tissue via its role in regulating oxidative stress, which contributes to chronic obstructive pulmonary disease (COPD) [21]. Moreover, other bioactive ingredients such as β-PAE, PAO, and pachypodol have attracted much attention in recent years, and the researches on their pharmacological effects as well as mechanisms has been deepened gradually. The chemical structures of PA, β-PAE, PAO, pogostone, and pachypodol are shown in Fig. 1. This review will provide detailed review of the mode of action for the selected chemical on major pharmacological activities.

To provide novel insights into the pharmacological mechanisms associated with patchouli, we conducted a search of the current literature using keywords including *Pogostemon cablin* Benth., patchouli, patchouli alcohol, patchouli oil, pogostone, patchoulene epoxide, beta-patchoulene, and bioactive compounds. We summarized the recent findings (2015–2020) focused on the phytochemistry, pharmacological activities and mechanisms of action of patchouli from studies published in PubMed, Web of Science, Springer, and the Chinese National Knowledge Infrastructure database. Initial search resulted in 171 studies but upon sorting based on the three themes (phytochemistry, pharmacological activities and mechanisms of action), our search yielded 62 studies that are featured in this review. Additional publications from years prior to 2015 were also included as they provide both insights and critical explanations .

##  Phytochemistry overview

*P. cablin* Benth. (patchouli) is an important aromatic plant that is native to southeast Asia and now cultivated widely in many tropical and subtropical regions, including China, Indonesia, the Philippines, and Thailand [22]. At present, the analysis and research on the chemical composition of patchouli mainly focus on the volatile oil, which is also known as patchouli oil (PO). Various ingredients have been reported in PO, such as PA, pogostone, α-guaiene, δ-guaiene, β-caryophyllene, trans-caryophyllene, α-patchoulene, β-patchoulene, and β-elemene [23]. Sesquiterpenes account for a large proportion of PO, and the content of PA is the highest among all the ingredients [24]. PA, a tricyclic sesquiterpene, can be isolated from the whole herb, stems, and leaves of patchouli by GC, GCMS, NMR, and other analytical methods [25, 26]. It possesses multiple bioactivities and is usually remarked as one of the indicators to distinguish the quality of PO. Besides, β-patchoulene and patchoulene epoxide are also the significant sesquiterpenes in PO, and the physicochemical properties as well as biological activities of these two substances have been reported in many researches [27–29]. Furthermore, pogostone is also abundant in patchouli, which is the effective ingredient of patchouli oil for insecticidal and antibacterial effects [30]. The detail information of some major and important volatile chemical components of patchouli oil are presented in Table 1. In addition to volatile oil, patchouli also contains a variety of non-volatile components with significant biological activity. So far, more than 50 non-volatile compounds have been discovered in patchouli and their chemical structures have been determined by various analytical methods [5]. These compounds can be roughly divided into terpenoids, flavonoids, glycosides, aldehydes, organic acids, and lignins according to their chemical structures. Some major and important compounds such as pachypodol, retusine, ombuin, apigenin, β-Sitosterol, stigmasterol, isocrenatoside, tilianin, 3ʺ-O-Methylcrenatoside, dibutyl phthalate, and tschimganical A have been reported in a number of researches [31, 32]. And among them, Pachypodol has attracted extensive attention due to its multiple biological activities [33, 34]. The information of the non-volatile compounds mentioned above are shown in Table 1.

**Table 1:** The chemical information of major components of patchouli

Chemical name	Formula	Type of compound	Molecular weight (g/mol)
Volatile components			
Patchouli alcohol	C_15_H_26_O	Sesquiterpenes	222.37
α, β-patchoulene	C_15_H_24_	Sesquiterpene	204.36
Patchoulene epoxide	C_15_H_24_O	Sesquiterpene	220.36
α, β-Guaiene	C_15_H_24_	Sesquiterpene	204.36
β-Caryophyllene	C_15_H_24_	Sesquiterpene	204.36
Trans-Caryophyllene	C_15_H_24_	Sesquiterpene	204.36
β-Elemene	C_15_H_24_	Sesquiterpene	204.36
Pogostone	C_12_H_16_O_4_	Pyrone	224.26
Non-volatile components			
Pachypodol	C_18_H_16_O_7_	Flavonoids	344.32
Retusine	C_19_H_18_O_7_	Flavonoids	358.35
Ombuin	C_17_H_14_O_7_	Flavonoids	330.29
Apigenin	C_15_H_10_O_5_	Flavonoids	270.24
β-Sitosterol	C_29_H_50_O	Phytosterols	414.72
Stigmasterol	C_29_H_48_O	Phytosterols	412.70
Isocrenatoside	C_29_H_34_O_15_	Glycosides	622.58
Tilianin	C_22_H_22_O_10_	Glycosides	446.41
3ʺ-O-Methylcrenatoside	C_29_H_36_O_15_	Glycosides	624.59
Dibutyl phthalate	C_16_H_22_O_4_	Organic Acids	278.35
Tschimganical A	C_11_H_16_O_3_	Others	196.25

**Fig. 2:**
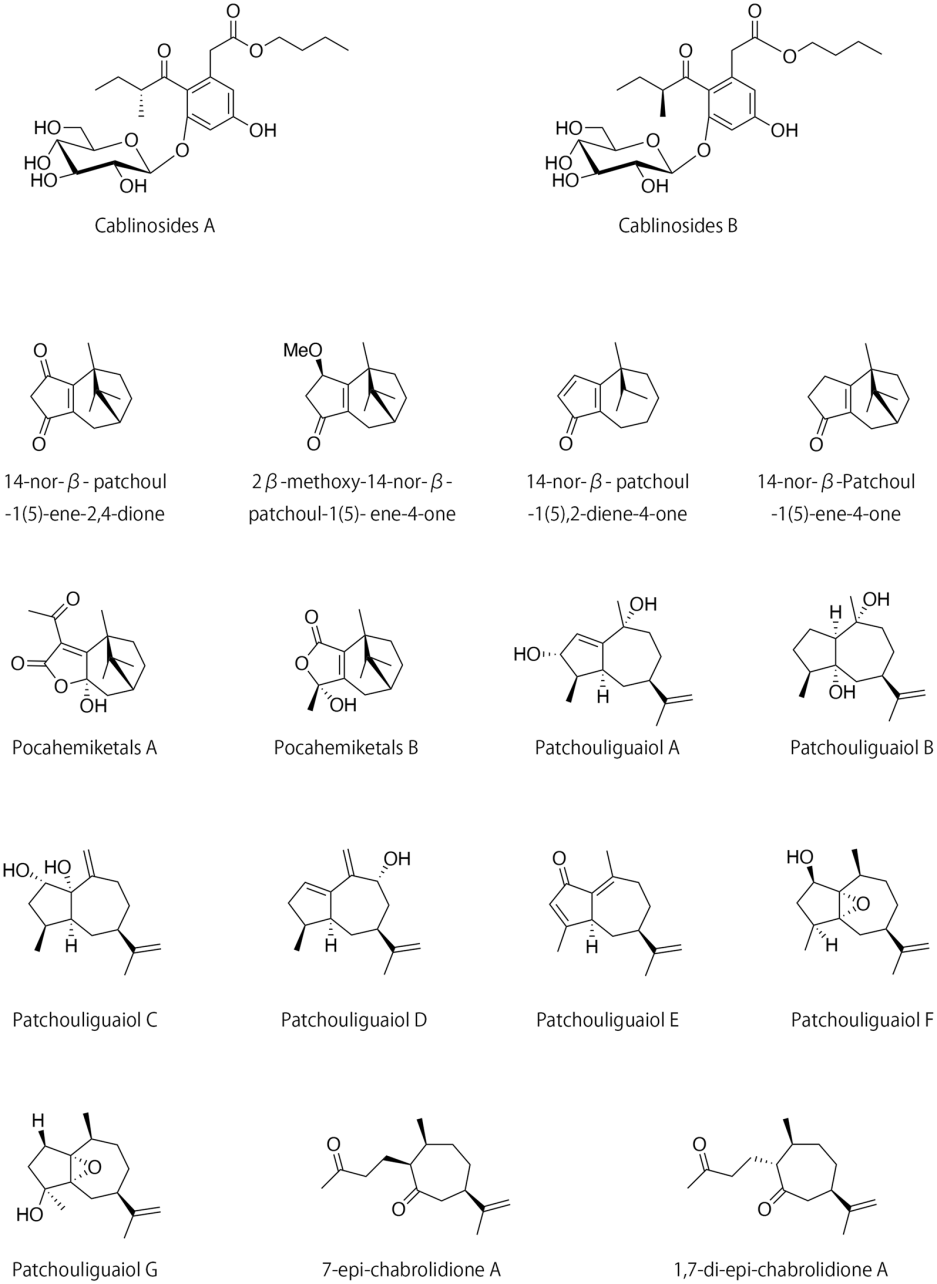
The chemical structures of new compounds in patchouli

Some novel ingredients of patchouli have been reported in studies from 2015 to 2020, and their chemical properties and pharmacological activities are included in Table 2. A Study in 2019 have described the identification and isolation of two new glycosidic epimers, cablinosides A and B, which were isolated from the leaves of *P. cablin* [35]. Their structures and associated absolute configurations were elucidated by nuclear magnetic resonance (NMR) and quantum chemical circular dichroism (CD) calculations. Pharmacological research demonstrated that the epimer mixture (including both cablinosides A and B) moderately inhibited the activity of the enzyme α-glucosidase, and was not toxic to human liver HepG2 cells. Similarly, four nor-β-patchoulene sesquiterpenoids were isolated from the essential oil of the leaves and stems of *P. cablin*; these include three new compounds, namely 14-nor-β-patchoul-1(5)-ene-2,4-dione, 2β-Methoxy-14-nor-β-patchoul-1(5)-ene-4-one, 14-nor-β-patchoul-1(5),2-diene-4-one and one new natural product 14-nor-β-Patchoul-1(5)-ene-4-one [36]. Their structures were elucidated by detailed spectroscopic analyses with one-dimensional (1D)- and two-dimensional (2D)-NMR techniques. Bioactivity testing revealed that 14-nor-β-patchoul-1(5),2-diene-4-one was slightly cytotoxic in assays that included both NCI-H1975 and HepG2 cells. Furthermore, another study have identified two novel hemiketal sesquiterpenoids that were isolated from the essential oil extracts from the aerial parts of patchouli [37]. The chemical structures of these novel compounds, pocahemiketals A and B, were determined by extensive spectroscopic analyses, electronic CD calculations, and single-crystal X-ray diffraction methods. Both pocahemiketals included a hemiketal α, a β-unsaturated-γ-lactone moiety, and a bicyclo[3.2.1]-carbon core; bioactivity assays revealed that Pocahemiketals B promoted significant vasorelaxant activity when tested against phenylephrine-induced contractions of a rat aorta ring at a half-maximal effective concentration (*EC*_50_) of 16.32 µM. In addition, seven novel guaiane sesquiterpenoids including Patchouliguaiol A-G and three previously characterized seco-guaianes were isolated from the volatile oil of patchouli; their structures were determined by spectroscopic analyses, a modified Mosher’s method, X-ray diffraction, and electronic CD data. Of these isolates, Patchouliguaiol C exhibited significant vasorelaxant activity (*EC*_50_ = 5.4 µM) when tested in assays of phenylephrine-induced contractions of rat aorta rings. Patchouliguaiol F was also characterized as a vasorelaxant with activity against phenylephrine- and KCl-induced contractions of rat aorta rings (*EC*_50_ of 1.6 and 24.2 µM, respectively). Notably, Patchouliguaiol C and Patchouliguaiol F also exhibited antifungal activity against *Candida albicans*, with minimum inhibitory concentrations (MICs) of 500 and 300 µM, respectively. In addition, Patchouliguaiol B, Patchouliguaiol G, 7-epi-chabrolidione A, and 1,7-di-epi-chabrolidione A exhibited neuroprotective effect in assays of glutamate-induced injuries targeting rat adrenal PC12 cells [38]. The chemical structures of the compounds mentioned above are presented in Fig. 2.

**Table 2:** The chemical properties and pharmacological activities of novel compounds

Compound name	Formula	Plant part	Analytical method	Type of compound	Bioactivity	References
Cablinosides A	C_23_H_34_O_10_	Leaves	HR-ESI-MS; UV; IR; NMR; HPLC; CD	Glycosides	α-glucosidase inhibitory activity (*IC*_50_ = 278.4 ± 2.8 µM)	[35]
Cablinosides B	C_23_H_34_O_10_	Leaves	HR-ESI-MS; UV; IR; NMR; HPLC; CD	Glycosides		[35]
14-nor-β-patchoul- 1(5)-ene-2,4-dione	C_14_H_18_O_2_	Leaves/Stems	TLC; NMR; IR; HR-ESI-MS	Sesquiterpenoids	–	[36]
2β-Methoxy-14-nor-β-patchoul-1(5)- ene-4-one	C_15_H_22_O_2_	Leaves/Stems	TLC; NMR; IR; HR-ESI-MS	Sesquiterpenoids	–	[36]
14-nor-β-patchoul- 1(5),2-diene-4-one	C_14_H_18_O	Leaves/Stems	TLC; NMR; IR; HR-ESI-MS	Sesquiterpenoids	Cytotoxic activities against NCIH1975 (*IC*_50_ = 49.9 µM)	[36]
					Cytotoxic activities against HePG-2 (*IC*_50_ = 56.0 µM)	
14-nor-β-Patchoul-1(5)-ene-4-one	C_14_H_20_O	Leaves/Stems	TLC; NMR; IR; HR-ESI-MS	Sesquiterpenoids	–	[36]
Pocahemiketals A	C_15_H_20_O_4_	Aerial parts	HR-ESI-MS; IR; NMR; X-ray; ECD	Sesquiterpenoids	–	[37]
Pocahemiketals B	C_14_H_20_O_3_	Aerial parts	HR-ESI-MS; IR; NMR; X-ray; ECD	Sesquiterpenoids	Vasorelaxant activity (*EC*_50_ = 16.32 µM)	[37]
Patchouliguaiol A	C_15_H_24_O_2_	Aerial parts	HR-ESI-MS; IR; NMR; ECD; X-ray	Sesquiterpenoids	–	[38]
Patchouliguaiol B	C_15_H_26_O_2_	Aerial parts	HR-ESI-MS; IR; NMR; ECD; X-ray	Sesquiterpenoids	Neuroprotective effect (50µM)	[38]
Patchouliguaiol C	C_15_H_24_O_2_	Aerial parts	HR-ESI-MS; IR; NMR; ECD; X-ray	Sesquiterpenoids	Vasorelaxant activity against PHE-induced contraction (*EC*_50_ = 5.4 µM)	[38]
					Antifungal activity against Candida albicans (*MIC* = 500 µM)	
Patchouliguaiol D	C_15_H_22_O	Aerial parts	HR-ESI-MS; IR; NMR; ECD; X-ray	Sesquiterpenoids	–	[38]
Patchouliguaiol E	C_15_H_20_O	Aerial parts	HR-ESI-MS; IR; NMR; ECD; X-ray	Sesquiterpenoids	–	[38]
Patchouliguaiol F	C_15_H_24_O_2_	Aerial parts	HR-ESI-MS; IR; NMR; ECD; X-ray	Sesquiterpenoids	Vasorelaxant activity against PHE- induced contraction (*EC*_50_ = 1.6 µM)	[38]
					Vasorelaxant activity against KCl- induced contraction (*EC*_50_ = 24.2 µM)	
					Antifungal activity against Candida albicans (*MIC* = 300 µM)	
Patchouliguaiol G	C_15_H_24_O_2_	Aerial parts	HR-ESI-MS; IR; NMR; ECD; X-ray	Sesquiterpenoids	Neuroprotective effect (50µM)	[38]
7-epi-chabrolidione A	C_15_H_24_O_2_	Aerial parts	NMR	Seco-guaianes	Neuroprotective effect (50µM)	[38]
1,7-di-epi-chabrolidione A	C_15_H_24_O_2_	Aerial parts	NMR	Seco-guaianes	Neuroprotective effect (50µM)	[38]

Generally, the chemical composition of medicinal materials is influenced by various factors, and geographic location is the most important one. In China, patchouli is mainly cultivated in Guangzhou city, Zhaoqing city, Zhanjiang city in Guangdong province, and some areas in Hainan province. According to its habitats, patchouli can be divided into *P. cablin cv.* Shipaiensis (also known as Paixiang), *P. cablin cv.* Gaoyaoensisensis (also known as Zhaoxiang), *P. cablin cv.* Zhangjiangensis (also known as Zhanxiang) and *P. cablin cv.* Hainanensis (also known as Nanxiang) [39], and traditional experience believes that Paixiang has the best quality. Previous studies illustrated that the difference in chemical composition of patchouli from diverse habitats was mainly characterized by the difference in the content of volatile oil. In 2006, 31, 33 and 42 volatile components were detected from Paixiang, Zhaoxiang and Zhanxiang, respectively using GC-MS. The extraction rates of essential oils of them were 0.25%, 0.40% and 0.64% respectively. Remarkably, the content of pogostone seems to be more susceptible to cultivation regions than other volatile components in patchouli, which can be summarized as the highest content of pogostone in Zhaoxiang, and the lowest content of pogostone in Paixiang [40]. In addition, the fingerprint of different solvent extraction sites of patchouli from Huangcun (Guangzhou City, Guangdong Province), Gaoyao (Zhaoqing City, Guangdong Province), Wuchuan (Zhanjiang City, Guangdong Province) and Wanning (Hainan Province) were compared and analyzed in 2011. The studies demonstrated that the similarity value of each extraction part of the water extract of Zhaoxiang and Paixiang was higher than 0.9, while the water extract of patchouli from Wuchuan, Wanning and Huangcun showed a large difference, which was consistent with the traditional experience for classifying patchouli by habitats. Moreover, the comparison of the fingerprints showed that the more polar components were less sensitive to cultivation regions [41]. In 2014, the content of PA and pogostone in patchouli from Leizhou County (Guangdong Province), Xuwen County (Guangdong Province), Yangchun County (Guangdong Province), Fumian County (Guangxi Province), and Wanning County (Hainan Province ) were determined and compared by GC. Based on the content of PA and pogostone, the results indicated that the quality of patchouli from Fumian was the best, followed by Yangchun, and the worst was Leizhou and Wanning [42]. It’s worth noting that the soil composition, climatic conditions and growth management of different cultivation regions may affect the properties and quality of the medicinal materials. Therefore, the quality control of patchouli and the research of medicinal resources are particularly important.

## Pharmacological activities

### Anti-peptic ulcer effect

Ulcers are detected primarily in the stomach and the proximal duodenum [43]; these lesions are the result of a multifactorial gastrointestinal disorder that has been associated with substantial morbidity and mortality, and affects many people worldwide [44]. Both endogenous and exogenous stimulation can contribute to the pathogenesis of this disease [45], most notably the chronic use of nonsteroidal anti-inflammatory drugs [46]. Moreover, pro-inflammatory cytokines, including tumor necrosis factor-α (TNF-α), interleukin-6 (IL-6), and interleukin-1 beta (IL-1β), as well as pro-apoptotic factors, are among the critical endogenous mediators that induce or aggravate GUs [47]. Pogostone, a characterized component of patchouli oil (PO), has been confirmed as effective against the oxidative stress associated with GUs [48]. Recent results have shown that pogostone can protect the gastrointestinal mucosa from indomethacin-associated GUs by its capacity to activate superoxide dismutase (SOD), glutathione (GSH), and catalase (CAT), and reduce the concentration of malondialdehyde (MDA) in rat models of disease. Levels of prostaglandin E_2_ (PGE_2_) and the protein and relative mRNA expression of cyclooxygenase 1 (COX-1) and cyclooxygenase 2 (COX-2) were all remarkably elevated in pogostone pretreated rats. The administration of pogostone also resulted in increased levels of heat-shock protein 70 and Bcl-2 protein, together with the diminished expression of Bax protein in ulcerated tissue [49]; these results indicated that pogostone was capable of suppressing cellular apoptosis as part of its gastroprotective mechanism. PAO, another component that has been isolated from PO, exhibits similar pharmacological effects; pretreatment with PAO significantly limited the extent of ethanol-induced GUs in rats. The administration of PAO resulted in increased levels of GSH, SOD, and CAT activities, together with the suppression of MDA in gastric tissues. Furthermore, PAO exhibited anti-inflammatory activity as it coordinated the production of both pro- and anti-inflammatory cytokines by its capacity to regulate the expression of several NF-κB pathway-related proteins. Moreover, immunohistochemistry revealed that the mechanism underlying PAO-mediated anti-apoptosis was largely related to its capacity to inhibit the expression of caspase-3, Fas, and FasL in the stomach tissue [50]. In addition to PAO, β-PAE mediates a prominent gastroprotective effect. A prominent metabolite of PA, β-PAE was significantly better than its parent compound at reducing the size of GUs in rats. Additionally, the administration of β-PAE resulted in dramatic reductions in the levels of MDA, TNF-α, IL-1β, and IL-6 in the serum, as well as the local expression of Fas, FasL, and caspase-3; the activities of SOD, GSH, and CAT were all increased concomitantly. The impact of β-PAE on GUs involved its interactions with both the NF-κB and ERK1/2 signaling pathways [51].

Inflammatory bowel disease (IBD) includes both ulcerative colitis (UC) and Crohn’s disease; both conditions are chronic and relapsing diseases of the gastrointestinal tract [52]. IBD includes severe gastrointestinal symptoms associated with the ulceration of the mucosa and submucosa of the colon and the rectum [53]; this disease is quite prevalent and affects a large number of individuals each year [54]. Previous research has revealed roles that both TNF-α and interleukins contribute to the pathogenesis of IBD [55]. The administration of PO reversed the colonic damage and reduced the disease activity indicators, including levels of colonic myeloperoxidase (MPO) in the 2,4,6-trinitrobenzenesulfonic acid-induced model of ulcerative colitis in rats [56]. Likewise, the administration of PA resulted in suppressed levels of colonic MPO as well as pro-inflammatory cytokines (i.e., TNF-α, IL-1β, and IL-6). The administration of PA also resulted in the suppression of several anti-inflammatory cytokines, including IL-4 and IL-10. UC-associated cellular pathology is dominated by the actions of activated Th2 cells, which mainly produce IL-4; likewise, IL-10 provides negative feedback during inflammation, as observed in studies featuring dextran sodium sulfate (DSS)-treated mice. The administration of PA also induces the expression of mRNA encoding mucin-1 and mucin-2 as well as the expression of the tight junction proteins that maintain the integrity of the intestinal epithelial barrier in mouse models of acute colitis. PA can also modulate the expression of apoptosis-related Bax and Bcl-2 proteins and thereby limit the pathology associated with DSS-induced signaling leading to cell death; PA can also downregulate the expression of the necrosis-associated protein, receptor-interacting protein kinase 3 [57]. Other studies [58] revealed that the PA-mediated activation of cytochrome P450 3A4 (CYP3A4) via a pregnane X receptor (PXR)-dependent mechanism resulted in attenuated inflammation via downstream signaling, which ultimately served to inhibit NF-κB activation and nuclear translocation; importantly, this study identified PA as a critical exogenous agonist of PXR. In vivo experiments revealed that PA prevented DDS-induced inflammation in mice by regulating PXR–NF-κB signaling. Taken together, these studies suggest that patchouli may have a profound impact on the pathogenesis of IBD mainly by its capacity to alleviate inflammation and modulate cellular apoptosis. Moreover, the identification of PA as a PXR agonist and a mediator of PXR–NF-κB signaling has provided insights into novel therapies that might be used to treat colitis. The pharmacological activities of patchouli with respect to peptic ulcer disease are included in Table 3.

**Table 3:** Pharmacological activities of patchouli on peptic ulcer

Chemical name	Animals & pathological model	Efficient doses & administration route	Mechanisms	References
Pogostone	SD Rats; Indomethacin-induced gastric ulcer	10, 20 and 40 mg/kg, oral administration	SOD↑, GSH↑, CAT↑, PGE2↑, COX-1↑, COX-2↑, HSP-70↑, Bcl-2↑, MDA↓, Bax↓	[48, 49]
PAO	SD Rats; Ethanol-induced gastric ulcer	10, 20 and 40 mg/kg, oral administration	GSH↑, SOD↑, CAT↑, IL-10↑, MDA↓, caspase-3↓, Fas↓, Fasl ↓, TNF-α↓, IL-1β↓	[50]
β-PAE	SD Rats, Ethanol-induced gastric injury	10, 20 and 40 mg/kg, oral administration	SOD↑, GSH↑, CAT ↑ MDA↓, TNF-α↓, IL-1β↓, IL-6↓, Fas↓, FasL↓, caspase-3 ↓	[51]
PO	SD Rats; 2,4,6-trinitrobenzenesulfonic acid-induced IBD	270 mg/kg, rectal instillation	MPO↓	[56]
PA	BalB/C mice; dextran sulfate sodium-induced colitis	10, 20 and 40 mg/kg, oral administration	MPO↓, TNF-α↓, IFN-γ↓, IL-1β↓, IL-6↓, IL-4↓, IL-10↓, ZO-1, ZO-2↑, claudin-1↑, occludin↑, mucin-1↑, mucin-2↑, Bax↓, Bcl-2↑, RIP3↓, MLKL↓, IDO-1↓, TPH-1↓,	[57]
	C57BL/6 mice; dextran sulphate sodium-induced colitis	6.25, 12.5 and 25 mg/kg, oral administration	CYP3A4↑, PXR ↑ NF-κB↓, IL-1β↓, IL-6↓, IL-10↓, TNF-α ↓	[48]

Arrow up denotes activation; arrow down denotes suppression

### Antimicrobial effect

#### Effect targeting *H. pylori*

**Fig. 3:**
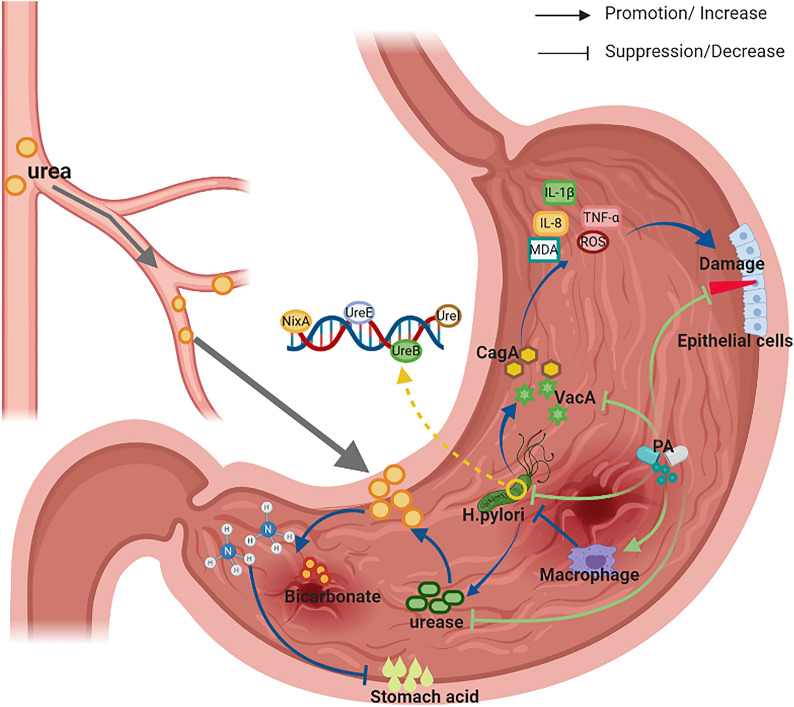
The pharmacological mechanisms of PA on H. pylori-induced gastric ulcer

*H. pylori* is a Gram-negative bacterial species that colonizes the gut of ~ 50% of the human population worldwide [59]; *H. pylori* has been associated with various gastrointestinal diseases including gastritis, peptic ulcers, and gastric cancer [60]. Previous research identified bacterial virulence factors that promote the pathogenesis of *H. pylori*-associated disease. Among the mechanisms that have been discovered, *H. pylori* produces urease, which hydrolyzes urea in peripheral circulation; this yields bicarbonate and ammonia that can counteract the acidic environment in the stomach [61]. However, *H. pylori* also releases pro-inflammatory toxins, such as vacuolating cytotoxin A (Vac A) and cytotoxin-associated gene A (Cag A); these toxins promote the release of pro-inflammatory cytokines that ultimately damage the epithelial cells in the gastric mucosa [62]. In addition, *H. pylori* can survive and persist within macrophages, as urease production serves to modulate the phagosome pH and the formation of megasomes [63]. PA is a critical pharmacological agent isolated from patchouli that exhibits antimicrobial activity against *H. pylori* both in vitro and in vivo. PA has selective antibacterial activity against *H. pylori*; it has no impact on the survival and proliferation of normal gastrointestinal bacteria and does not promote bacterial resistance. The administration of PA limits the adhesion and motility of *H. pylori*, and inhibits the expression of critical bacterial genes together with host inflammatory mediators [64]. PA has been shown to inhibit the activity of urease protein in both acidic and neutral conditions by blocking both protein maturation [65] and the pathway that facilitates the translocation of Ni^2+^, which eventually decrease the acid resistance of this bacterial strain [66]. PA at 25 and 50 µM can inhibit intracellular *H. pylori*-associated urease activity by downregulating the expression of genes encoding ureB, ureE, ureI, and nixA; this reduces the UreB protein level and thus facilitates macrophage-mediated antimicrobial activity [67]. PA also promotes direct cytoprotective effects and limits the damage to epithelial cells associated with persistent *H. pylori* infection. Recent studies [68] revealed that PA could also reverse the cytotoxicity for gastric epithelial cells (GES-1) that results from an overabundance of *H. pylori*-associated urease. Specifically, the administration of PA effectively attenuated GES-1 apoptosis by actions that support the integrity of the mitochondrial membrane potential, which attenuate oxidative stress by decreasing the contents of intracellular reactive oxygen species (ROS) and MDA, and which promote the synthesis and activation of both SOD and CAT. As such, PA serves as an anti-inflammatory agent by eliminating *H. pylori* and inhibiting the expression of bacterial virulence factors and also by its actions that modulate signaling via the NF-κB and NLRP3 inflammasome activation pathways [69]. PA can also reduce *H. pylori*-mediated neutrophil recruitment and activation by inhibiting the production of pro-inflammatory cytokines, by its actions that target p22 and p47-phox, as well by modulating the expression of the *H. pylori* neutrophil activation-related gene [70]. Furthermore, PA can eradicate *H. pylori* and limit oxidative stress by blocking bacterial escape from the intracellular lysosome compartment [71]. The pharmacological mechanisms used by PA to target *H. pylori*-induced GU are shown in Fig. 3. Taken together, these results suggest that patchouli extracts may be useful for the treatment of GU activities by their capacity to inhibit oxidative damage, reverse inflammation, induce apoptosis-associated signaling pathways, and eliminate the *H. pylori* pathogen.

#### Other antimicrobial effect

Earlier studies identified numerous antimicrobial activities associated with the use of patchouli [7]; among the microorganisms identified in these studies, patchouli promotes resistance against *Staphylococcus aureus*, *Vibrio harveyi*, and *Moraxella catarrhalis*. Aqueous extracts of patchouli effectively inhibit biofilm formation associated with multidrug-resistant *V. harveyi* by inducing the upregulation of the biofilm-related bacterial genes luxR and flaB, and by downregulating the expression of luxS, hfq, and ompW with an MIC of 31.25 mg/mL [72]. In addition, PO has antimicrobial activities against both Gram-positive and Gram-negative microorganisms, with an MIC of 25 mg/mL against isolates of *Streptococcus mutans*, and 12.5 mg/mL against both *Shigella flexneri* and *S. aureus* in studies carried out in vitro [73]. Likewise, the patchouli constituent, pogostone, exhibited significant antibacterial effect on *S. aureus* in experiments carried out in vitro with an MIC of 4 µg/mL. Pogostone may exert its antibacterial effect by interacting with *S. aureus* cell membrane proteins and its capacity to alter cell membrane permeability [74]. Acute otitis media, one of the most common diseases in early infancy and childhood [75], is a common bacterial complication of viral upper respiratory tract infection [76]. In vitro experiments revealed that PO was active against both *S. aureus* and *M. catarrhalis* with MICs of 0.21 and 0.026 mg/mL, respectively. The administration of PO promotes the resolution of *S. aureus* and *M. catarrhalis* infections in the middle ear and reduces the extent of inflammatory cell infiltration at the middle ear mucosa [77].

### Anti-oxidative effect

Oxidative stress refers to increased levels of intracellular ROS that result in damage to lipids, proteins, and DNA [78]; this is the negative effect associated with the production of free radicals in vivo [79]. Oxidative stress can result from numerous external and endogenous factors [80], including alcohol, drugs, and environmental pollutants; these factors eventually promote premature aging and the negative sequelae of severe diseases [81]. NF-E2-related factor-2 (Nrf2) is a transcription factor that activates antioxidant response elements to regulate the expression of a variety of cytoprotective genes, including detoxifying, antioxidant, and antiapoptotic proteins [82]. Previous studies revealed that PO exhibits significant antioxidant potential, by actions that promote the elimination of superoxide anion free radicals and hydroxyl free radicals, and that inhibit lipid peroxidation [83]. In addition to the nonvolatile constituents of *P. cablin*, the administration of pachypodol can attenuate ROS production and thereby protect hepatocytes from oxidative cell death induced by tert-butyl-hydroperoxide. The underlying mechanism of this effect relates to the amplification of the endogenous antioxidant defense system via the ERK1/2-dependent activation of Nrf2 [34]. Alcohol intake can also promote oxidative damage to the liver, as it increases the activity of cytochrome P450 2E1 (CYP2E1) and leads to the generation of large quantities of ROS; this ultimately destroys the oxidation/reduction balance maintained by GSH/GSSG [84, 85]. Ethanol-induced acute liver injury, typically caused by excessive alcohol consumption, has been associated with several serious liver disorders including alcoholic fatty liver, hepatitis, hepatic fibrosis, steatosis, and cirrhosis [86]. Hepatic steatosis has been widely recognized as an early and reversible consequence of excessive alcohol consumption [87]; pretreatment with PO results in the increased concentration of the hepatic antioxidant, GSH, and a concomitant increase in the GSH/GSSG ratio, as well as the activation of anti-oxidative enzymes glutathione reductase (GR) and SOD. These responses serve to suppress the accumulation of ROS and decrease the expression of protein and mRNA encoding CYP2E1. The administration of PO could also prevent fatty degeneration by its capacity to accelerate adipose metabolism [88]. Similar to what is observed in response to biochemical oxidative stress, heat shock-induced oxidative stress may promote damage to and apoptosis of intestinal epithelial-6 cells (IEC-6) [89]. Pretreatment with PA circumvents the damage to the cellular morphology and results in a decrease in the MDA content that accumulated secondary to heat shock. High doses of PA also resulted in significant increases in the expression of Nrf2 and HO-1. Taken together, these results demonstrated that PA was capable of mitigating cell damage and alleviating the oxidative stress responses of IEC-6 cells by the activation of the Nrf2-Keap1 pathway [90].

Aging of the skin, induced by both intrinsic and extrinsic factors, is associated with a gradual loss of structural integrity and physiological function [91]. Skin photoaging is related to the increased activity of matrix metalloproteinases (MMPs) that were induced in response to the production of ROS [92]. MMP-mediated alterations in the extracellular matrix typically lead to skin wrinkling, which is a prominent feature of premature aging [93]. The administration of pogostone alleviates both the macroscopic and histopathological lesions observed in UV-damaged skin in mouse model systems; it promoted the activities of the antioxidant enzymes, including CAT, SOD, and GSH-PX, downregulated MDA levels, and inhibited aberrant expression of MMP-1 and MMP-3 [94]. In addition, PO had a substantial therapeutic impact on photoaged rat skin by its capacity to regulate p38MAPK/ERK and the associated apoptotic signaling pathway. Recent studies [95] revealed that the administration of PO prevented aberrant increases in MDA, p38MAPK, Ras, Raf, mitogen-activated protein kinase (MEK), ERK1/2, Bax, Caspase9, c-Fos, and c-Jun, as well as the aberrant decreases in Bcl2, SOD, GSH-PX, and CAT. As such, we conclude that patchouli-mediated anti-photoaging activities may be associated with its anti-oxidative, anti-inflammatory, and antiapoptotic properties.

### Anti-inflammatory effect

Inflammation is characterized by redness, swelling, heat, and pain at one or more affected locations; this process represents a primary protective response against invading pathogens [96]. These responses are mediated by circulating pro-inflammatory mediators, including IL-6, IL-1β, TNF-α, nitric oxide (NO), and PGE_2_, among others [97]. As such, agents capable of regulating inflammation by the synthesis and release of pro-inflammatory mediators are great significance as a means to control this response. Lipopolysaccharide (LPS) is a major component of Gram-negative bacteria that mediates inflammation initiated by macrophage responses [98]. β-PAE exhibits significant anti-inflammatory effect on LPS-stimulated RAW 264.7 macrophages by its capacity to maintain the balance between pro- and anti-inflammatory cytokine production [99]. Pretreatment with β-PAE results in significantly diminished levels of TNF-α, IL-6, and IL-1β biosynthesis, accompanied by an increased expression of IL-10. β-PAE also suppressed inducible nitric-oxide synthase (iNOS) and COX-2 signaling pathways, resulting in decreased levels of NO and PGE_2_. NF-κB signaling is central to the development and progression of acute inflammation; its activation and translocation is required to promote the transcription of many pro-inflammatory mediators. NF-κB is a hetero-tetramer comprised of two proteins known as p65 and p50. In the latent state, NF-κB is sequestered in the cytosol in association with its inhibitor, IκB (inhibitor of NF-κB); the induction of an inflammatory response destroys this balance, which results in proteasomal degradation and the nuclear translocation of NF-κB [100]. β-PAE inhibits the translocation of NF-κB from the cytoplasm to the nucleus and stabilizes the cytoplasmic nuclear factor-κBα (IκBα) complex [101]. The actions of β-PAE also promote a decrease in the levels of both MDA and MPO activity in association with edema, and suppress the activation of pro-inflammatory cytokines including TNF-α, IL-6, IL-1β, PGE_2_, and NO in a dose-dependent manner in mouse models. Given its high degree of structural similarity to β-PAE, it was not surprising to find that aged preparations enriched in PAO also exhibit anti-inflammatory effect. The administration of PAO resulted in decreased levels of IL-1β, IL-6, TNF-α, PGE2, and NO, and concomitant increased levels of IL-4 and IL-10. PAO also promoted the downregulation of both protein and mRNA encoding COX-2 and iNOS, and limited the activation of NF-κB signaling pathways by its capacity to inhibit the translocation of p50 and p65 from the cytosol to the nucleus [102]. Interestingly, recent studies revealed that PAO was superior to β-PAE with respect to their capacities to limit inflammation. As the oxidative product of β-PAE, PAO exerted potent anti-inflammatory activities in vivo, which included the decreased expression of both protein and mRNA encoding TNF-α, IL-12, IL-1β, and monocyte chemotactic protein-1 (MCP-1). In addition, the anti-inflammatory effect of PAO were superior to those of β-PAE in experiments that examined production of NO and PGE2 via their corresponding iNOS and COX-2 signaling pathways [103]. The anti-inflammatory effect of PO were also examined; this agent served to limit leukocyte recruitment by its capacity to interfere with the production and activation of NO and pro-inflammatory cytokines [104]. The pharmacological activities of patchouli with respect to anti-inflammatory responses are presented in Table 4.

**Table 4:** Pharmacological activities of patchouli in anti-inflammation

Chemical name	Animals/cells and Pathological model	Type	Efficient doses and administration route	Results	References
β-PAE	LPS-stimulatedRAW264.7 macrophages	In vitro	10, 20, 40 μmol/L	TNF-α↓, IL-6↓, IL-1β↓, IL-10↑, iNOS↓, COX-2↓, NO↓, PGE2↓	[99]
	Kun Ming (KM) mice; Xylene-induced ear edema, Acetic acid-induced vascular permeability, Carrageenan-induced paw edema	In vivo	10, 20, 40 mg/kgoral administration	MDA↓, MPO↓, TNF-α↓, IL-1β↓, PGE2↓, IL-6↓, NO↓, iNOS↓, COX-2↓, p65 (nuclear)↓	[100]
PAO	KM mice; Xylene-induced ear edema, Acetic acid-induced vascular permeability, Carrageenan-induced paw edema	In vivo	10, 20, 40 mg/kg oral administration	TNF-α↓, IL-1β↓, IL-6↓, PGE2↓, NO↓, IL-4↑, IL-10↑, COX-2↓, iNOS↓, p-IKKβ and IκBα↓	[102]
	LPS-stimulated RAW264.7 macrophages	In vitro	10, 20, 40 μmol/L	TNF-α↓, IL-12↓, IL-1β↓, MCP-1↓, PGE2↓, NO↓, iNOS↓, COX-2↓	[103]
PO	Swiss mice; Zymosan-induced peritonitis	In vivo	100, 200, 300 mg/kg oral administration	Leukocyte recruitment↓, NO↓, leukocyte number↓	[104]
	fMLP-induced neutrophils	In vitro	1, 3, 10, 30, 60, 90 mg/ml	Neutrophil migration↓	
			3, 10 mg/ml	Phagocytic activity of neutrophils↑	

Note: Arrow up denotes activation; arrow down denotes suppression

### Effect on ischemia/reperfusion (I/R) injury

I/R injury is associated with several serious clinical manifestations, including acute heart failure, gastrointestinal dysfunction, myocardial hibernation, cerebral dysfunction, systemic inflammatory response syndrome, and multiple organ dysfunction [105]; the last condition is associated with an extraordinarily high mortality rate and requires timely treatment to protect the brain from injury [106]. Inflammation is a critical feature of cerebral I/R injury. The specifics associated with the inflammatory response determine the extent and nature of the brain damage that may ensue; these factors are connected with several signaling pathways, including the MAPK and the Toll-like receptor 4 (TLR4)/NF-κB signaling pathways, among others. Therefore, it is critical to suppress the inflammatory response associated with I/R. Toward this end, the results of several studies revealed that the administration of PA could reduce infarct volume and alleviate the ensuing blood–brain barrier dysfunction in a model of obese mice with cerebral I/R injury. Levels of protein and mRNA encoding TNF-α and IL-1β were diminished in response to the administration of PA, together with diminished phosphorylation of JNK and p38; these results demonstrate that PA can provide protection against cerebral I/R injury by its capacity to inhibit inflammatory responses [15]. Interestingly, cell apoptosis and oxidative stress also contributed to the development and progression of I/R injury. In addition to its capacity to alleviate inflammation by inhibiting TLR4/NF-κB signaling, pretreatment with β-PAE also results in a significant suppression of cellular apoptosis in I/R injury in rats, largely by decreasing the Bax/Bcl-2 expression ratio and limiting the induction of caspase-3 activity. Elevated levels of glutathione peroxidase (GSH-PX) and SOD were detected, while superoxide generation and MDA levels were reduced [107].

### Analgesic effect

Pain is initiated by the activation of various nociceptors via specific stimulus modalities. Pain is a common symptom of many diseases and has an outsized impact on normal life and physiological homeostasis. Patchouli directly inhibited the impact of acetic acid-induced writhing (pain) in mice; these results implied that patchouli exhibits an analgesic effect in vivo [108]. COX-2, an inflammatory cyclooxygenase, is induced in response to pro-inflammatory cytokines at sites of inflammation; this enzyme is often upregulated in response to inflammation and in association with neoplastic disease [109]. The administration of PA upregulated COX-2 mRNA and protein expression both in vivo and in vitro. The anti-nociceptive impact of PA involves the mu-opioid receptor (MOR) [18]. Opioids are highly effective analgesics; opioid systems are critical with respect to pain regulation, pain-associated behavior, and pain relief [110]. MOR upregulation has a direct impact on intracellular calcium concentrations by the activation of calcium channels; as such, the calcium ion concentration can be utilized as a marker to study the role of PA vis-à-vis the function of MOR [111]. Recent studies suggest that PA could simultaneously upregulate MOR expression in the mouse brain and decrease intracellular calcium levels; this was not observed in response to the administration of aspirin. As such, the role of patchouli with respect to its capacity to modulate the activation of both COX-2 and MOR may provide a significant basis for further studies of PA as a new form of analgesic.

### Antitumor effect

Several recent studies provided results that elucidated patchouli-mediated antitumor activity; the underlying mechanisms have been revealed to some extent. Recent results revealed that an aqueous extract of patchouli could overcome the resistance of endometrial cancer cells to paclitaxel and could likewise promote growth inhibition [112]. PA also exhibited antitumor effect when targeting cells of the human lung cancer A549 line both in vitro and in vivo by activating both caspase 9 and caspase 3 and modulating mitochondria-mediated apoptosis; the underlying molecular mechanism involves inhibited phosphorylation of EGFR and the phosphorylation of targets within the JNK signaling pathway [113]. The administration of PA can also inhibit the proliferation of human leukemia MV4-11 cells and thereby induce their apoptosis; the mechanisms underlying this response may be related to a decrease in NF-κB and phospho-pyruvate kinase M2 (p-PKM2), and the increase of Caspase-3 protein expression [114]. Pogostone is another antitumor constituent of patchouli; recent studies [20] revealed that pogostone could inhibit the proliferation and the colony formation of gallbladder carcinoma SGC-996 cells by its capacity to promote the expression of caspase-9, caspase-3, and poly-ADP-ribose polymerase-1 (PARP-1), to increase the Bax/Bcl-2 ratio, and to decrease the expression of cyclin D1, cyclin A, and cyclin B. Taken together, these findings suggest that the antitumor effect of pogostone may be related to the regulation of apoptosis- and cell cycle-regulated proteins.

### Antidiabetic effect

Obesity is highly correlated with incidence of type 2 diabetes and a primary risk factor for various metabolic diseases. It is a factor contributing to the condition known as metabolic syndrome; this condition is exacerbated by environmental factors, including a fat-enriched diet, a sedentary lifestyle, and potentially by aging [115]. The administration of PA resulted in a net decrease in body weight of high-fat diet (HFD)-induced obese mice; PA suppressed adipogenesis and fat accumulation in adipocytes by increasing the expression and activation of beta-catenin [116]. The chronic intake of a HFD has also been associated with numerous other diseases, including NAFLD. NAFLD is a major cause of liver disease that affects ~ 30% of the US population [117], and is currently the most common chronic liver disorder worldwide [118]. Recent studies [14] have explored the protective effects of PA when used to treat HFD-induced hepatic steatosis in rats; these studies demonstrated that PA was effective in ameliorating hepatic steatosis resulting from a HFD. PA mediated this effect by suppressing endoplasmic reticulum stress signals and by regulating the uptake, assembly, and secretion of very low-density lipoproteins. Among the underlying mechanisms considered, the administration of PA is also associated with the regulation of the very low-density lipoprotein receptor, apolipoprotein B100, as well as microsomal triglyceride-transfer protein expression.

### Anti-hypertensive effect

Hypertension is a chronic and critical factor that promotes disability and can lead to premature death [119]. More than one billion individuals worldwide carry a diagnosis of hypertension; this condition is associated with ~ 9.4 million deaths each year [120]. Agents capable of reducing systemic blood pressure can significantly reduce the risk of events associated with major cardiovascular disease, including stroke and coronary heart disease, among others [121]. As such, it is particularly important to maintain blood pressure within a normal range. PA promotes significant vasorelaxant effect as a result of its role as a Ca^2+^ antagonist in an endothelium-independent pathway. The underlying mechanisms include the blockade of extracellular Ca^2+^ influx via the membrane of vascular smooth muscle cells and the release of intracellular Ca^2+^ through IP3R- and RYR-mediated Ca^2+^ channels in the sarcolemma [19]. Pocahemiketal B isolated from the essential oil generated from the aerial parts of *P. cablin* exhibited significant vasorelaxant activity against phenylephrine-induced contractions of rat aorta rings, with an *EC*_*50*_ of 16.32 µM [37].

### Immunoregulatory effect

The immune system plays a vital role in maintaining the integrity of an organism; the immune system mediates both resistance to pathogens and defense against cancer [122]. Previous research revealed that PA has a positive effect on the immune system, and can promote immunomodulatory actions by activating the mononuclear phagocytic system and by suppressing overactive cellular immune responses [123]. There are also recent reports of PO-mediated immunomodulatory activities; these are associated mainly with its capacity to promote the synthesis and release of secretory immunoglobulin A (SIgA). SIgA is a first-line immune defense of the surface of the intestinal mucosa; SIgA antibodies promote mucosal immunity, which includes host defense against food antigens, bacteria, viruses, and toxins [124]. The administration of PO promotes te repair of the intestinal epithelial ultrastructure, reduces intestinal permeability, and protects the intestinal mucosal mechanical barrier in a rat model of post-infectious IBS; the underlying mechanisms include promoting increased levels of SIgA while inhibiting the expression of ICAM-1 [125].

### **Effect on intestinal microecology**

The appropriate balance of the gut microbiota (GM) is of great importance for human health. The GM extract nutrients and energy [126], protect us from enteropathogens [127] and cancer [128], and may even influence brain function and behavior [129]. Irregularities of the GM, a state known as dysbiosis, may be a predisposing factor associated with IBD [130, 131], obesity [132, 133], and neoplastic disease [134]. The results of several studies have suggested that PO and its derivatives, including pogostone, PA, and β-PAE, serve to support the function of the gut epithelial barrier, to facilitate the polarization of M1 to M2 macrophage phenotypes, to increase the diversity of the GM, and to suppress the pro-inflammatory cytokines in mouse model systems. Taken together, these results suggest that the pharmacological activities of PA, pogostone, and β-PAE contribute to the dynamic interactions between the host and the GM [135].

### **Antidiarrheal effect**

Irritable bowel syndrome (IBS) is a common functional bowel disorder; diarrhea-predominant IBS (IBS-D) is a major subtype of this disease [136]. At least one study has demonstrated that PA exhibits a concentration-dependent inhibitory effect on spontaneous contractions of the colonic longitudinal smooth muscle, with an *EC*_*50*_ of 41.9 µM [137]. PA also promoted the inhibition of IBS-D as modeled in the rat colon by actions associated with cholinergic, nitrergic, and K^+^ channel pathways. These results demonstrated that PA might be the active element underlying the antidiarrheal activity of patchouli, although the pharmacological targets of these effects remain unknown.

### **Other effects**

Secretory otitis media (SOM) includes inflammation of the mucosa of the middle ear, and is characterized by tympanic effusion, ear tightness, and hearing loss; these responses are typically associated with bacterial infection and can eventually lead to auditory tube dysfunction [138]. Recent studies revealed that pogostone could reverse the hearing loss typically associated with SOM in experiments performed in a guinea pig model; the administration of pogostone alleviated the thickening of the mucous membrane and neutrophil infiltration by its capacity to inhibit the expression of TNF-α and intercellular cell adhesion molecule (ICAM)-1 in the mucous membranes of the ear [139]. In addition, lung inflammation has been associated with several serious respiratory diseases, including acute respiratory distress syndrome and COPD, among others. The administration of PA serves to protect against LPS-induced acute lung injury in mice by the suppression of TNF-α, IL-1β, and IL-6 synthesis and release, as well as by its capacity to inhibit the phosphorylation of IκB-α and p65 NF-κB. The overall mechanism underlying the PA-mediated inhibition of the inflammatory response could be attributed to the inhibition of the NF-kB signaling pathway [140]. Moreover, pogostone could exert protective effect with respect to lung injury associated with COPD, also in a mouse model; pogosone suppressed the expression of inflammatory-related proteins (p-IκBα and p-NF-κBp65) and promoted a significant increase in Nrf-2 and HO-1. Overall, these results suggested that the pogostone-mediated inhibition of the NF-κB signaling pathway could be the central mechanism underlying the protection of pulmonary tissue [21]. Inflammatory cytokines, including iNOS, TNF-α, and the interleukins, all promote the pathogenesis of atherosclerosis. Atherosclerosis is a chronic disease of the arterial wall [141]; the disorder is characterized by lipid deposition and the formation of foam cells in the vessel intima. Recent studies have illustrated that the administration of PA resulted in a significant attenuation of atherosclerotic plaques both in the aorta and at the aortic root; PA also resulted in the elimination of macrophages from the cell contents of lesions in atherosclerosis-prone apolipoprotein E knockout (ApoE KO) mice. Moreover, PA inhibited the expression of aortic-associated macrophage inflammatory cytokines, such as IL-1β, iNOS, MCP-1, IL-6, and chemokine (C-X-C motif) ligand 11 in mouse model systems; these results demonstrated that PA could promote the attenuation of atherosclerosis, possibly by inhibiting macrophage infiltration and its inflammatory responses [16].

## Toxicity

Given the widespread interest in and application of TCM throughout the world, reports of adverse reactions and adverse events have been increasing; this has generated significant concern regarding the toxicities associated with TCM and TCM-associated medicinal preparations. Previous experiments carried out in mice revealed that PA was associated with comparatively low toxicity; the lethal dose (*LD*_*50*_) of PA was determined to be 4.7 g/kg when administered via intragastric administration and 3.1 g/kg in response to intraperitoneal injections [142]. Recently, PO and its major components (PA and pogostone) exerted significant toxicity with respect to the development of zebrafish embryos; among the findings, these agents were associated with an increased incidence of notochord malformation as well as cardiac and yolk edema in zebrafish larvae, with the toxicity of pogostone > PA > PO. The 50% lethal concentrations (*LC*_*50*_*s*) of PA and pogostone were 50.3 and 12.9 mg/L, respectively, determined at 24 h after administration; the *LC*_*50*_s of PO, PA, and pogostone were 21.2, 12.9, and 11.8 mg/L, respectively, at 96 h after administration [143]. Although patchouli has been used for > 2000 years in China, our current understanding of systemic toxicity and safety remain inadequate; these points require much additional study and careful evaluation.

## Conclusion and future research

In recent time, herbs and extractions from TCM, as well as derivatives, are gaining acceptance as potentially promising complementary and alternative medicines for various diseases treatment [144–146]. The plant family Labiatae (Lamiaceae) is famous for its outstanding medicinal and aromatic herbs, which is a rich source of essential oils for the food, pharmaceutical and cosmetic industry [147]. In addition to patchouli, several other herbs such as *Agastache rugosa*, *Elsholtzia ciliata* (Thuab) Hyland., *Leonurus japonicas* Houtt., and *Perilla frutescens* (L.) Britt. also possess kinds of bioactivities and can be used to treat diseases. Among the diverse herbs in the Labiatae family, *Agastache rugosa* (*A. rugosa*), a medicinal plant of Labiatae genus’s Agastache rugosa (Fisch. et Mey.) O. Ktze., has a lot of characteristics that are similar to patchouli. *A. rugosa*, commonly known as “Tuhuoxiang” or “Chuanhuoxiang” in China, is native to Sichuan Province, Jiangsu Province, and Zhejiang Province. As an edible plant, it is used as a herbal medicine to treat nausea, vomiting and dispel damp in TCM [148]. *A. rugosa* was reported to have prominent pharmacological activities, such as anti-gastritis effect [149], anti-photoaging effect [150], anti-melanogenesis effect [151], anti-microbial effect [152], anti-tumor effect [153], anti-oxidant effect [154], and anti-atherogenic effect [155]. Although most of its pharmacological effects are similar to that of patchouli, its main chemical components are quite different. In 2013, a total of 88 chromatographic peaks were separated from the volatile oil of *A. rugosa*, and 45 compounds were identified. Among these compounds, isopulegone, pulegone, as well as isomenthone account for a large proportion, while PA and pogostone are the major components of patchouli [156]. In addition, the microscopic identification of *A. rugosa* and patchouli also showed some differences. For instance, two cells formed the head of glandular hairy in the leaves of patchouli, while only one formed the head of glandular hairy in the leaves of *A. rugosa*. Moreover, the nonglandular hair was mainly constituted by one to three cells in patchouli, while one to four cells constituted the nonglandular hair in *A. rugosa* [157].

**Fig. 4:**
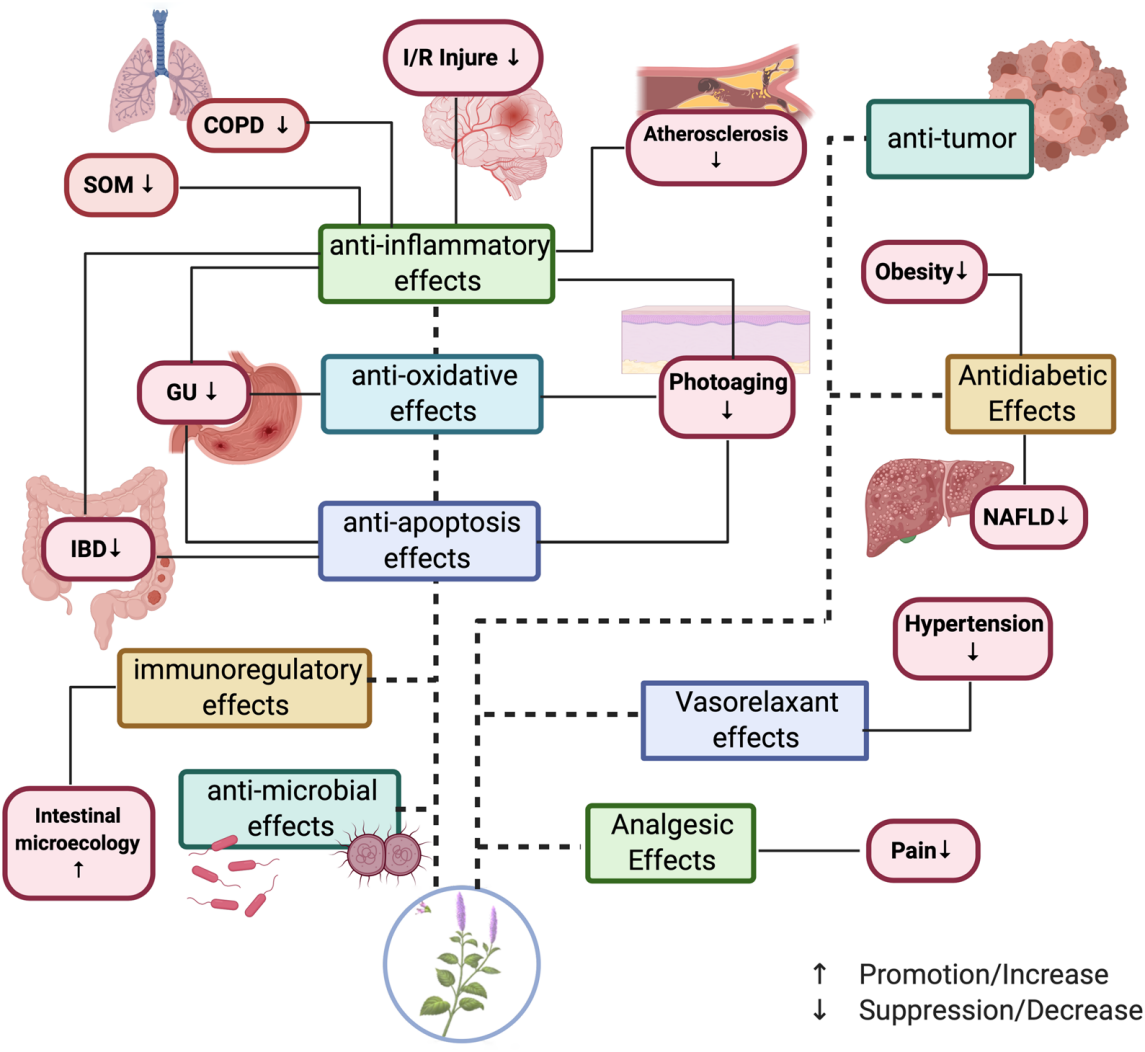
The pharmacological activities of patchouli

**Fig. 5:**
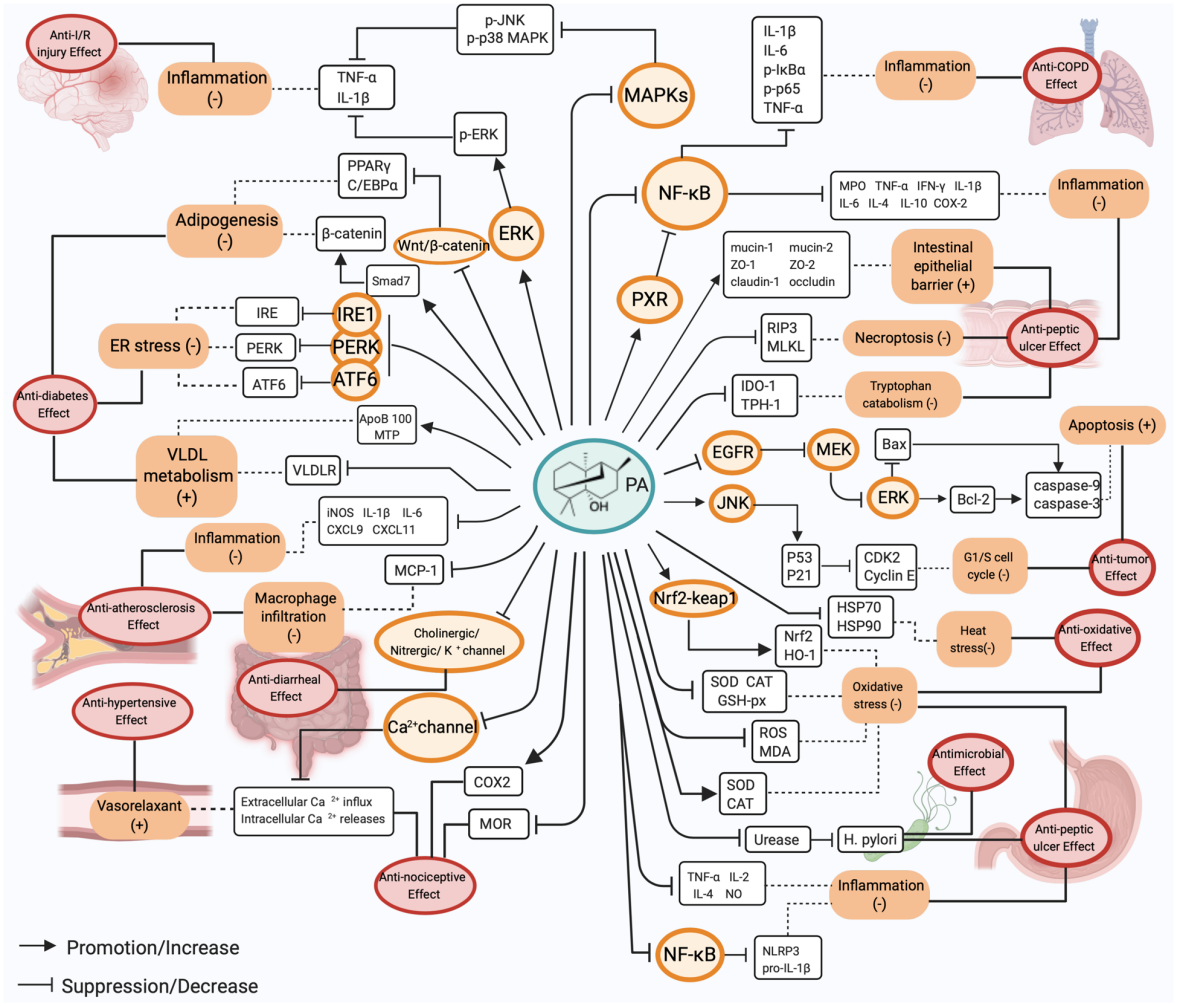
The molecular and cellular targets of PA

*P. cablin* Benth., commonly known as patchouli, is an important medicinal herb with huge market potential in the fragrance industry; it is also the main ingredient in numerous Chinese patent medicines. Comprehensive experimental research studies performed in the past five years have complemented the pharmacological activities and mechanisms of action of patchouli, including anti-peptic ulcer effect, antimicrobial effect, anti-oxidative effect, anti-inflammatory effect, I/R injury protection, analgesic effect, antitumor effect, antidiabetic effect, anti-hypertensive effect, immunoregulatory effect, effect on intestinal microecology, antidiarrheal effect and others. Results from multiple studies demonstrated that patchouli and its derivatives can promote protective effects on the stomach, intestines, liver, and even the middle ear. The pharmacological activities of patchouli noted above are featured in Fig. 4. With the development of research, the monomeric components in patchouli such as PA, β-patchoulene, patchoulene epoxide, pogostone, as well as pachypodol have been explored to some extent, and the pharmacological mechanisms study of PA is the most profound. The molecular and cellular targets of PA mentioned in this review are portrayed in Fig. 5. From these studies, we can conclude that the therapeutic actions of PA are related to its capacity to suppress inflammation, alleviate oxidative stress, regulate apoptosis, relieve ER stress, increase VLDL metabolism, and others. Further investigations focusing on the molecular mechanisms indicated that multiple signalling pathways are involved in the treatment process. The PA-mediated relief of peptic ulcer is associated with the activation of PXR signalling pathway, the inhibition of the NF-κB pathway, the preservation of intestinal barrier integrity, the suppression of tryptophan catabolism, and the inhibition of cell death signaling [57, 58]. The modulations of the these pathways result in the decrease of pro-inflammatory cytokines, the downregulation of the necroptosis related RIP3 and MLKL proteins, the downregulation of the IDO-1 and TPH-1 protein, the downregulation of pro-apoptotic protein Bax, and increase of the anti-apoptotic protein Bcl-2. In addition, the PA-mediated treatment of diabetes is related to the inhibition of the PERK, IRE1, ATF6, and Wnt/β-catenin pathways, which eventually results in the downregulation of PERK, IRE1, and ATF6, the inhibition of VLDLR, the increase of apoB 100, the enhancement of MTP, the increased expression of smad7, and the stabilization of β-catenin [14, 17]. Moreover, by activating the ERK signaling pathway and inhibiting the MAPKs pathway, PA plays a important role in the treatment of I/R injury [15]. Furthermore, other pathways such as the EGFR pathways, JNK pathways, Nrf2-keap1 pathways, as well as Cholinergic channel, Nitrergic channel, K^+^ channel, and Ca^2+^ channel also participate in the therapeutic actions of PA [16, 18, 19, 90, 113]. The novel pharmacological effects associated with PA, for instance, its role in preventing obesity and promoting analgesia, have attracted significant attention in recent years, although the underlying mechanisms remain unclear. Likewise, more researches are needed to elucidate the molecular mechanism of action and protein targets of other ingredients in patchouli.

In addition, our current understanding of the critical safety factors of patchouli is somewhat inadequate; additional preclinical studies that feature both acute and long-term toxicities associated with patchouli should be carried out in the near future. Pharmacological studies of patchouli and its derivative compounds have been performed primarily in vitro and in vivo using small animal models. As such, clinical studies in humans are urgently needed to confirm these pharmacological findings and to promote the development of TCM preparations for extended use worldwide.

## Data Availability

Not applicable.

## Abbreviations

PA: Patchouli alcohol
β-PAE: β-patchoulene
PAO: Patchoulene epoxide
β-PAO: β-patchoulene epoxide
PEO: Patchouli essential oil
PO: Patchouli oil
GU: Gastric ulcer
NLRP3: NACHT, LRR, and PYD domain-containing protein 3
NO: Nitric oxide
PGE2: Prostaglandin E2
TNF-α: Tumor necrosis factor-α
IL-6: Interleukin-6
IL-1β: Interleukin-1 beta
iNOS: Inducible nitric-oxide synthase
COX-2: Cyclooxygenase
MOR: Mu-opioid receptor
p-PKM2: Phospho-pyruvate kinase M2
PARP-1: Poly-ADP-ribose polymerase-1
MMP: Matrix metalloproteinase
ROS: Reactive oxygen species
CAT: Catalase
SOD: Superoxide dismutase
MDA: Malondialdehyde
MEK: Mitogen-activated protein kinase
ERK1/2: Extracellular-regulated kinase 1/2
GSH-PX: Glutathione peroxidase
Nrf2: NF-E2-related factor-2
HPU: Helicobacter pylori urease
GES-1: Gastric epithelial cells
Glu: Glutathione
IBD: Inflammatory bowel disease
DDS: Dextran sodium sulfate
IBS: Irritable bowel syndrome
IBS-D: Diarrhea-predominant irritable bowel syndrome
IEC-6: Intestinal epithelial-6 cells
GM: Gut microbiota
I/R: Ischemia/reperfusion
MCP-1: Monocyte chemotactic protein 1
CXCL11: Chemokine (C-X-C motif) ligand 11
NAFLD: Nonalcoholic fatty liver disease
LPS: Lipopolysaccharide
TCM: Traditional Chinese medicine
